# Toward a computational theory of manifold untangling: from global embedding to local flattening

**DOI:** 10.3389/fncom.2023.1197031

**Published:** 2023-05-31

**Authors:** Xin Li, Shuo Wang

**Affiliations:** ^1^Lane Department of Computer Science and Electrical Engineering (CSEE), West Virginia University, Morgantown, WV, United States; ^2^Department of Radiology, Washington University at St. Louis, St. Louis, MO, United States

**Keywords:** blessing of dimensionality, object recognition, motor control, manifold embedding, manifold flattening

## Abstract

It has been hypothesized that the ventral stream processing for object recognition is based on a mechanism called cortically local subspace untangling. A mathematical abstraction of object recognition by the visual cortex is how to untangle the manifolds associated with different object categories. Such a manifold untangling problem is closely related to the celebrated kernel trick in metric space. In this paper, we conjecture that there is a more general solution to manifold untangling in the topological space without artificially defining any distance metric. Geometrically, we can either *embed* a manifold in a higher-dimensional space to promote selectivity or *flatten* a manifold to promote tolerance. General strategies of both global manifold embedding and local manifold flattening are presented and connected with existing work on the untangling of image, audio, and language data. We also discuss the implications of untangling the manifold into motor control and internal representations.

## 1. Introduction

Is dimensionality a curse or a blessing? The term “curse of dimensionality” was coined by Richard Bellman when studying dynamical programming in the 1960s (Bellman, 1966). It refers to various phenomena that arise from the analysis and organization of data in high-dimensional spaces. Specifically, all objects tend to become sparse and dissimilar in many ways as the dimensionality increases, which prevents common data organization strategies from being efficient. To overcome such a curse of dimensionality, various non-linear dimensionality reduction techniques such as IsoMAP (Tenenbaum et al., 2000) and locally linear embedding (LLE) (Roweis and Saul, 2000) have been developed to reveal the low-dimensional structure embedded in high-dimensional observation data.

The blessing of dimensionality (Donoho, 2000) is a more counter-intuitive concept. To illustrate this concept, we start by considering a classical toy example of XOR decision for the linear perceptron (Rosenblatt, 1958). There is no 2D linear classifier that can separate the two different classes of XOR decision. However, with an additional dimension *z* = *x* ⊕ *y*, it is straightforward to linearly separate two classes in a 3D space (*x, y, z*) (e.g., hyperplane $z=\frac{1}{2}$ will do). Another example of so-called two-circle data consisting of two concentric circles, each representing a different class. Again, there exists no linearly classifier that can separate red from blue in 2D; while linear separability can be easily satisfied in 3D by taking into account the third and redundant dimension $r=\sqrt{x^{2}+y^{2}}$ into account.

We note that the issue of dimensionality is often tangled with that of linearity. For example, Kernel trick (Schölkopf, 2000) in support vector machine (SVM), which allows linear learning algorithms to learn a non-linear function or decision boundary, can be interpreted as a special class of techniques exploiting the blessing of dimensionality. In face verification (Chen et al., 2013), linear feature dimension as large as 100K has been reported to improve performance due to the blessing of dimensionality. More recently, the class of convolutional neural networks, equipped with non-linear rectifying linear units (ReLU), has shown excellent performance in various vision tasks from image classification to object recognition. Between non-linearity and dimensionality, which plays a more fundamental role?

In this paper, we advocate for the blessing of dimensionality from a manifold untangling perspective (Chung and Abbott, 2021). The problem of manifold untangling (a.k.a. disentanglement, Brahma et al., 2015) can be formulated as an extension of the manifold embedding and knotting problem (Skopenkov, 2008) in differential topology. Originating from Whitney's original work in 1930 (Whitney, 1936), blessing-of-dimensionality related results include embedding of the *n*-manifold in *R*^2*n*^ and unknotting in *R*^2*n*+1^ (Wu, 2008). These classical results in the theory of differential topology inspire us to tackle the problem of manifold untangling by iteratively constructing overparameterized direct-fit models (Hasson et al., 2020) in a higher-dimensional space. The main contributions of this paper are summarized below.

- Manifold untangling without a distance metric. In topological space, we show how to improve the manifold capacity by a unified untangling approach.
- Two general strategies for untangling manifolds: global embedding vs. local flattening. We show how embedding and flattening jointly improve manifold capacity by promoting selectivity and tolerance.
- Model-agnostic for multimodal data. We apply the theory of manifold untangling to several recent works on multiview image recognition, invariant audio recognition, and perceptual video straightening.
- Biological connection with the hypothesis of cortically local subspace untangling in ventral stream processing and trajectory untangling in motor control.

## 2. Manifold untangling: what and why?

### 2.1. Problem formulation

**Figure 1:**
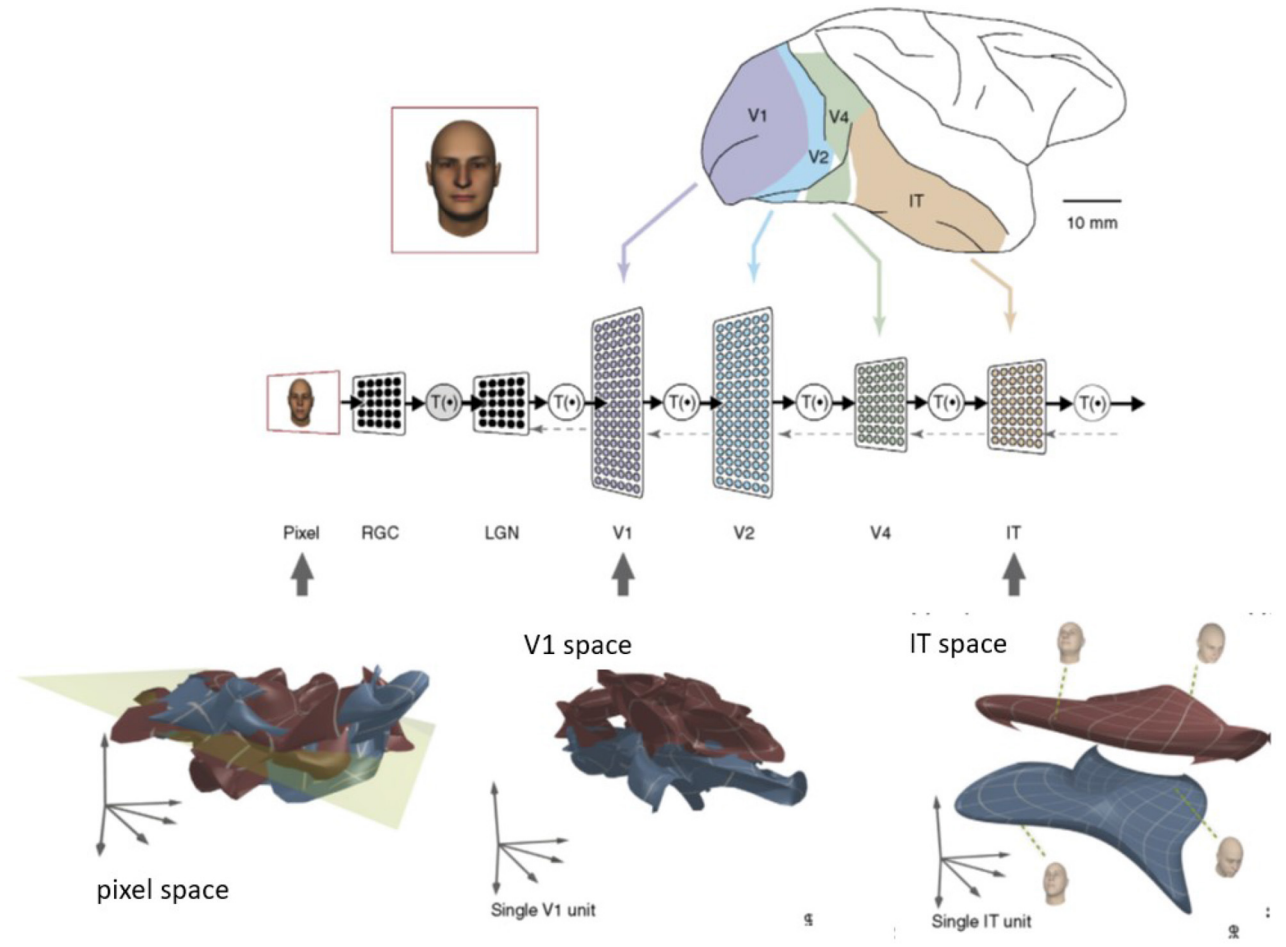
Illustration of manifold untangling by neuronal population along the ventral visual processing stream (DiCarlo and Cox, 2007). This figure was created from the published paper in TRENDS in Cognitive Sciences, Vol. 11, No. 8, James J. DiCarlo and David D. Cox, Untangling Invariant Object Recognition, 333–341. Copyright Elsevier (2007).

The problem of manifold untangling originated from the modeling of ventral stream processing in neuroscience (DiCarlo and Cox, 2007) (see Figure 1). To explain how object recognition works, a major challenge is the form of high-dimensional visual representations. An object manifold (e.g., the image projected onto the retina) is characterized by variations of its pose, position, and size, which can be mathematically abstracted as a low-dimensional curved surface inside the retinal image space. It follows that different objects, such as varying face identities, correspond to different manifolds. The term “object manifold” specifically refers to low-dimensional subspaces underlying population activities embedded in high-dimensional neural state space according to Chung and Abbott (2021). The manifolds embedded in the ambient neural state space (called the neural population geometry in Chung and Abbott, 2021) include both sensory/motor and cognitive regions of the brain.

To illustrate the problem of manifold untangling more vividly, we can use an analogy with tangled shoelaces in our familiar 3D Euclidean space. The task of object recognition is analogous to untangle these shoelaces but in a higher-dimensional space of visual representations. In the literature, manifold untangling (a.k.a. disentanglement, Brahma et al., 2015) has also been studied for other data modalities, such as image (Cohen et al., 2020), speech (Stephenson et al., 2019), video (Hénaff et al., 2019), and language (Mamou et al., 2020). There are two conflicting objectives for manifold untangling (DiCarlo et al., 2012): promoting selectivity (i.e., to separate two manifolds associated with different identities/objects) and boosting tolerance (i.e., to achieve invariance to pose, position, scale, and cluttered background). Selectivity and tolerance are closely related to the two types of errors (false alarm and miss detection) in pattern recognition. The fundamental hypothesis behind our approach is that nature has discovered a clever solution to manifold untangling in the topological space which the need of defining a distance metric.

### 2.2. Motivation: topological space does not require a distance metric

One of the long-standing open problems in manifold discovery is how to calculate the geodesic distance between two points on a manifold. Unlike the Euclidean distance, the geodesic distance is intrinsically tangled with the locally curved low-dimensional geometry of the manifold. Without knowledge of local geometry, calculating the geodesic distance or building a kernel becomes a tangled problem like manifold learning (Ma and Fu, 2012). Can one solve the problem of untangling a manifold without discovering its local low-dimensional structure? Does there exist a universal solution to manifold untangling by global operations such as homotopy (Hatcher, 2005)?

We argue that the answer is affirmative. Our basic intuition is based on the observation that it is easier to untangle a manifold in a higher-dimensional space (Fusi et al., 2016). A simple justification is based on the observation that a knot in three dimensions can be untied when placed in a four-dimensional space (Crowell and Fox, 2012). More generally, in higher dimensions than four, there is enough “space” to untie any knot by smoothly transforming it into a circle. Recent studies on unsupervised disentanglement of manifold (Horan et al., 2021) show that local isometry (related to embedding) and non-Gaussianity (required by linear generative models) make disentanglement possible. Both conditions are more easily satisfied in higher-dimensional spaces.

To quantify the effectiveness of manifold untangling, the manifold capacity (Chung et al., 2018) has been derived from the mean-field theoretic analysis. The basic idea is to find the maximum number of dichotomies that are linearly separable in a high-dimensional space. Conceptually, manifold capacity can be enhanced by promoting selectivity (e.g., pushing object manifolds away from each other) or boosting tolerance (e.g., smoothing rugged surfaces of object manifolds). More rigorously, there are two complementary approaches to maximize the manifold capacity: manifold embedding (promoting selectivity) in a higher-dimensional space and manifold flattening (boosting tolerance) to facilitate linear separability. The main question lies in the construction of embedding or flattening functions to increase the manifold capacity, as we will elaborate next.

## 3. Manifold embedding and flattening

### 3.1. Manifold embedding and unknotting theory

**Theorem 1. Whitney Embedding Theorem (1936)**.

Any smooth manifold **M** of dimension *m* ≥ 2 can be embedded into *R*^2*m*+1^.

In 1958, W.T. Wu proved that every connected *n*-manifold unknots in *R*^2*n*+1^ for *n* > 1 (Wu, 2008). The theory of differential manifold was extended into surgery theory by J. Milnor in the 1960s, which became a major tool in high-dimensional topology. An important class of smoothing manifolds was to use obstruction theories (Hirsch, 1963). Obstruction theory is concerned with when a topological manifold has a piecewise-linear structure and when a piecewise-linear manifold has a differential structure.

**Figure 2:**
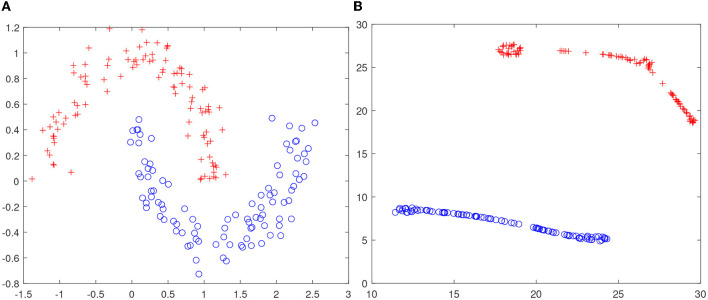
Blessing of dimensionality. **(A)** Two-moon data are not linearly separable in *R*^2^; **(B)** t-SNE visualization of the LLE embedding in *R*^4^. Note that two-moon data becomes linearly separable after embedding in a higher-dimensional space *R*^4^ through locally linear embedding (LLE) (Roweis and Saul, 2000).

The intuition that higher-dimensional space facilitates the task of manifold untangling has not been well-documented in the literature. The closest result seems to be (Tauro et al., 2014). To shed some insight to the blessing of dimensionality, we have conducted a simple experiment with the synthetic two-moon data (see Figure 2A). It is easy to observe that these data are not linearly separable in *R*^2^; however, we have verified that after locally linear embedding (LLE) (Roweis and Saul, 2000), a linear dichotomy exists, as shown in Figure 2B. Note that unlike kernel trick in support vector machine, we do not resort to non-linearity but the blessing of dimensionality for a data representation that is less tangled.

Based on the above line of reasoning, the basic ideas behind our approach to maximize the manifold capacity in a higher-dimensional space are as follows. On the one hand, we want to increase the number of distinct manifolds by promoting the *selectivity* of data representations (i.e., pushing more manifolds away from each other). This objective can be achieved by embedding the manifold into a higher-dimensional space using the generalized kernel trick such as LLE or IsoMAP (Tenenbaum et al., 2000) (note that we use them in the opposite direction to non-linear dimensionality reduction—i.e., as the tools of non-linear dimensionality increase). On the other hand, we want to increase the number of separable dichotomies by promoting *tolerance* of data representations. This is aligned with the idea of manifold flattening by constructing identity-preserving transformations (DiCarlo et al., 2012) or smoothing the decision boundaries (Verma et al., 2019). Both global embedding and local flattening contribute to the objective of manifold untangling, but in a complementary manner.

### 3.2. Global manifold embedding

At the global level (i.e., working with the entire manifold as a whole), there are two broad classes of manifold embedding techniques: kernel methods and sparse coding. Both of them can re-represent input data in a higher-dimensional space to facilitate the task of manifold untangling.

#### 3.2.1. Recursive and generalized kernel methods

**Figure 3:**
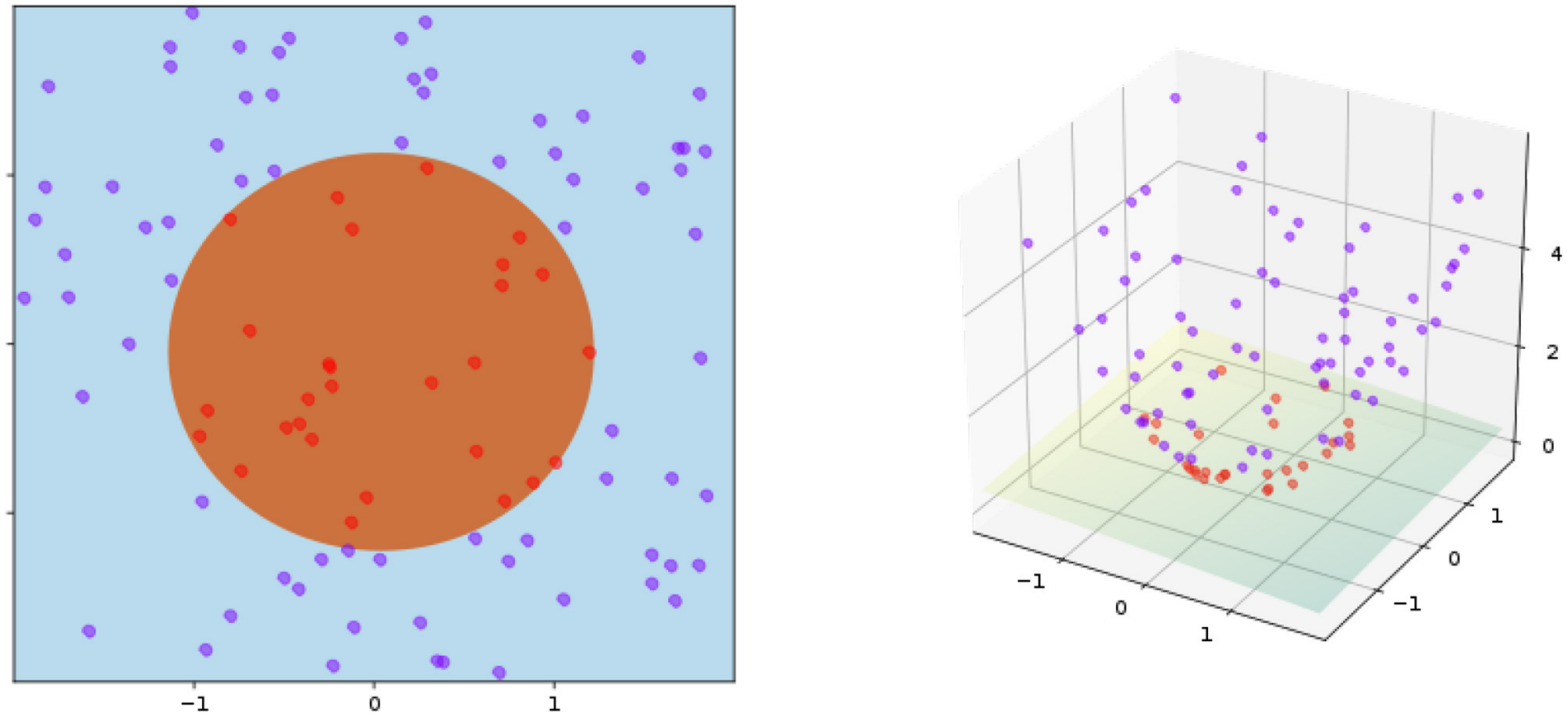
Kernel trick in the inner product space (**left**: input space, **right**: feature space). The kernel is given by ϕ((*a, b*)) = (*a, b, a*^2^ + *b*^2^) and *K*(***x***, ***y***) = ***x*** · ***y***+ ∥ ***x*** ∥^2^ + ∥ ***y*** ∥^2^. Training points are mapped to a 3-dimensional space, where a separate hyperplane can be easily found.

A well-known method, named the kernel trick, is to generalize distance-based algorithms to operate in the feature space (Schölkopf, 2000). The key idea is to construct a non-linear mapping function ϕ : **X** → **Y** where ***x*** ∈ **X** and ϕ(***x***) ∈ **Y** denote the input and feature spaces, respectively. Then, the kernel trick is implemented by the dot product in the feature space, i.e., *k*(***x***, ***x***′) =< ϕ(***x***), ϕ(***x***′) >. For the class of positive definite kernels, rigorous results, such as Mercer's theorem (Vapnik, 1999) guarantees the generalization of distance metric for a wide range of kernel constructions (e.g., radial basis function and neural tangent kernel). As a concrete example, Figure 3 illustrates the idea behind the kernel trick for a toy example of separating points within a circle from those outside.

The effectiveness of the kernel trick is often attributed to its non-linearity related to the input space. However, dealing with non-linearity is always challenging—e.g., despite the conceptual simplicity of the kernel trick, it is often much more difficult to reason with the optimality of different approaches to kernel construction. More importantly, as shown in Figure 3, the blessing of dimensionality offers a refreshing perspective to understand the kernel trick. The new dimension introduced by the kernel geometrically warps the data points in such a way that they can be more easily separated by a linear classifier. Such a simple observation inspires us to tackle the manifold untangling by recursively applying the kernel trick.

More specifically, we propose to generalize the non-linear mapping function ϕ : **X**^*n*^ → **X**^*n*+1^, *n* ∈ *N*, where ***x***^*n*^ ∈ **X**^*n*^ and ϕ^*n*^(***x***^*n*^) ∈ **X**^*n*+1^, *dim*(**X**^*n*+1^) > *dim*(**X**^*n*^) denote the input and output spaces in the *n*-th layer, respectively. Our intuition is that manifold untangling is closely related to the approximation by non-linear sigmoid functions (Cybenko, 1989).

**Theorem 2. Universal Approximation Theorem**.

For any continuous function *f*(*x*) and sigmoidal function σ, there exists a universal approximation by $g\left(x\right)=\sum_{j=1}^{N}\alpha_{j}\sigma \left(y_{j}^{T}x+\theta_{j}\right)$ such that |*f*(*x*) − *g*(*x*) < ϵ| for all *x* ∈ *I*_*n*_, where *I*_*n*_ denotes an n-dimensional unit cube.

The approximation result above can be interpreted as the untangling of the non-linear function *f*(*x*) by successive concatenation of *N* sigmoid unit in a single hidden layer. Each unit partially untangles the non-linear function until the input function is straightened into a linear one. Connecting this result with our manifold untangling intuition, we can interpret multilayer feedforward networks as universal approximators (Hornik et al., 1989) that recursively untangle a non-linear function (decision region) until we reach the linear separable regime.

#### 3.2.2. Hierarchical sparse coding

The equivalence relationship between the kernel method in a support vector machine (SVM) (Bartlett and Shawe-Taylor, 1999) and sparse coding (Olshausen and Field, 1997) has been well-studied in the literature (Girosi, 1998). An important new insight brought about by this work is the generalization of kernel trick by hierarchical sparse coding. As advocated in DiCarlo et al. (2012), the organized hierarchy forms a closed loop from primary visual cortex (V1) to inferior temporal cortex (IT) and then back to V1. The hierarchical organization is reflected by the increasing field-of-view, as well as improved tolerance of IT population to object recognition. An intuitive explanation for such hierarchical organization is that it leads to a redundant but sparse representation that promotes the selectivity of visual stimuli.

More rigorously, we consider the class of hierarchical and redundant sparse representations [e.g., steerable pyramids (Simoncelli and Freeman, 1995) and overcomplete dictionaries (Olshausen and Field, 1997)] from the perspective of manifold embedding. They map the retinal image space to a much higher dimensional space with sparse coefficients. Unlike the non-linearity argument supplied by Olshausen and Field (1997), we argue that exploiting the blessing of dimensionality plays a more fundamental role in not only V1 but also the entire processing of the ventral stream. Note that this is consistent with H. Barlow's redundancy exploitation hypothesis (Barlow, 2001) because the sparse coding strategy maximizes the capacity of associative memory (Olshausen and Field, 2004).

Under the framework of manifold untangling, we claim that hierarchical sparse coding increases the number of manifolds (manifold capacity) while keeping the feature dimension (*N*) constant. In view of the lack of a rigorous definition of manifold capacity in the literature, we resort to a closely-related concept (the capacity of associative memory) in our analysis. A mathematical analysis of why sparse coding increases the capacity of associative memory can be found in Okada (1996). It was shown that the sparsely coded associative memory model achieves an extremely large storage capacity that diverges as the mean-firing rate decreases. Despite the increase in the total number of coefficients in redundant sparse representation, it is easy to observe that the *ratio* of significant coefficients (effective dimensionality of salient features corresponding to the mean firing rates) does not change due to the good localization properties of bases.

To show how improved sparsity increases the capacity of associative memory, we consider a non-holographic associative memory model in Willshaw et al. (1969) which consists of *N*_*A*_ × *N*_*B*_ grid points on a square lattice. Let $r_{A}=\frac{M_{A}}{N_{A}}$ and $r_{B}=\frac{M_{B}}{N_{B}}$ denote the ratio of active grid points responsible for the associative recall of *R* cross-link patterns. Then, the memory capacity of such an associative network is given by


(1) $$\begin{array}{l} C=N_{c}\cdot log\left(p\right)\cdot log\left(1-p\right), \end{array}$$

where *N*_*c*_ = *N*_*A*_ × *N*_*B*_ and the collision probability *p* can be calculated by


(2) $$\begin{array}{l} 1-p=exp\left(-R\cdot r_{A}\cdot r_{B}\right), \end{array}$$

It is easy to observe that to maintain a low collision probability *p*, both *r*_*A*_ and *r*_*B*_ need to be small, implying a small percentage of active grid points along the horizontal and vertical directions. The improvement in sparsity in the representation of the data helps reduce the probability of collision (less crosstalk) (Olshausen and Field, 2004) by promoting the selectivity of the associative representations. Note that the above 2D toy model (square lattice) can easily be generalized to a high-dimensional integral lattice *Z*^*n*^. Sparser representations can reduce the probability of collision, leading to much increased capacity of associative networks. In the literature on neurobiology, high-dimensional representations with mixed selectivity (Fusi et al., 2016) have been shown to allow for linear separable decision regions to support different potential responses.

### 3.3. Local manifold flattening

At the local level (i.e., dealing with the local geometry of a manifold), we can smooth either the rugged surface underlying the data observations or the curved decision boundaries separating different classes.

#### 3.3.1. Identity-preserving transformations

The other important new insight deals with the discovery of local geometry on a manifold to promote tolerance within the same class/identity. The importance of tolerance to object recognition can be mathematically justified by flattening the manifold with identity-preserving transformations (see Figure 2B in DiCarlo et al., 2012). More specifically, consider the curved surface of an object manifold (e.g., projection onto a subspace) associated with position or scale; achieving tolerance (i.e., translation or scale invariance) is conceptually equivalent to unfurling the curved surface such that the flattened manifolds can be more easily separated by hyperplanes. Some quantitative evidence to validate the flattening hypothesis in deep learning has been reported in Brahma et al. (2015).

The manifold untangling framework offers a refreshing perspective on the well-studied binding problem (Treisman, 1996). After manifold flattening, each untangled subspace is characterized by the neural population geometry, whose representation simultaneously conveys explicit information about not only object identity but also tangled subspace attributes such as position, size, pose, and context. Even when multiple objects are present, one can imagine that identity-preserving transformations can flatten their corresponding manifolds to improve the manifold capacity. There is no need to rebind those subspace attributes because they are implicitly embedded into identity-preserving transformations.

To better illustrate the concept of manifold flattening, we can think of the three pairs of legs in jacks as an analogy to the identity, position, and scale subspaces. Mathematically, these jacks can be interpreted as a 1D manifold embedded into a 3D Euclidean space. The problem of packing object manifolds is challenging because the legs of those jacks interfere with each other. Identity-preserving transformations facilitate the packing task by flattening the two subspaces of position and scale (we will discuss the biological implementation of this strategy later). In the transformed space after manifold untangling (i.e., conditioned on the knowledge about the position and scale), the jacks are flattened to ellipsoids suitable for packing or linear separation.

#### 3.3.2. Decision boundary smoothing

An alternative approach to achieve the objective of local manifold flattening is via smoothing the decision boundary among different classes/identities. Along this line of reasoning, several closely related ideas have recently been proposed such as manifold mixing (Verma et al., 2019), manifold charting (Mangla et al., 2020), and embedding propagation (Rodríguez et al., 2020) and have been shown to be effective for few shot classification.

The objective of manifold flattening is to reduce the number of directions with significant variance (refer to Figure 2B). Following the notation in Verma et al. (2019), we use X,H,Y to denote input space, representation space, and output space, respectively. The representation space can be the hidden states of DNN or support vectors of SVM or sparse coefficients in hierarchical sparse coding. We can obtain the following theoretical result.

**Theorem 3. Manifold Flattening Theorem**.

Let H be a space of dimension dim(H), and let *d* represent the number of classes/identities in the dataset. If dim(H)≥d-1, then there exists a linear function/dichotomy that can separate the *d* different classes.

The proof of the above result for the hidden state of the DNN representations can be found in Verma et al. (2019). Generally speaking, if the dimensionality of the representation dim(H) is greater than the number of classes *d*, then the resulting representations for that class will fall into a subspace of dimension dim(H)-d+1.

It is enlightening to compare the boundary smoothing strategy of decision with that of identity-preserving transformations. The former improves the performance of the classifier in the presence of distribution shifts, outliers, and adversarial examples with few-shot learning constraint (i.e., it does not require much training data). The latter requires more training data to achieve the desired objective of X-invariant recognition (X refers to environmental uncertainty factor) by learning identity-preserving transformations. These two approaches are complementary to each other because they flatten the manifold from different (inter-class vs. intra-class) perspectives.

## 4. Model-agnostic manifold untangling

### 4.1. Multi-view visual object recognition

Visual object recognition has been extensively studied by the computer vision community (Zhang et al., 2013; Bakry and Elgammal, 2014). The three subspaces associated with object category, instance, and viewpoint/pose are often tangled in the observation of multiview image data. Conventional wisdom to achieve an untangled representation of the view-object manifold is to formulate a joint reconstruction problem with unknown category/instance and viewpoint. Through parameterization of the visual manifold by a mapping coefficient matrix and a non-linear kernel map, one can formulate a continuous inference problem (Zhang et al., 2013) or a discrete discrimination problem (Bakry and Elgammal, 2014). Therefore, the objective of manifold untangling is implicitly implemented by projecting onto the target subspace of category, instance, and viewpoint.

A fundamental weakness of those conventional approaches is their lack of generalization property. It is often assumed as a priori that the topology of the viewpoint manifold of individual objects is known. The derived manifold untangling solution easily breaks down when such an assumption becomes invalid (e.g., due to the tangling of other uncertainty factors such as scale, illumination, and clutter, Johnson and Hebert, 1999). Meanwhile, the computational complexity of manifold reconstruction in both continuous and discrete settings can be prohibitive because of the required Monte-Carlo Markov-Chain (MCMC) sampling and exhaustive search of subspace indexes (the curse of dimensionality). One cannot help wondering if there exists an explicit solution to manifold untangling without reconstruction.

This work offers attractive alternative solutions to multiview visual object recognition. In several challenging datasets with the presence of pose and expression variations, it has been shown in Chen et al. (2013) that high-dimensional features (as large as 100K) can dramatically boost face verification performance. This blessing of dimensionality has been empirically verified for various local descriptions from local binary patterns (LBP) (Ahonen et al., 2004) to Gabor filters (Liu and Wechsler, 2002). Our manifold embedding strategy offers a plausible theoretical interpretation—namely, as the dimensionality increases, the concatenation of features with varying landmark numbers and sampling scales promotes selectivity by offering complementary descriptions of the object category.

Identity-preserving transformations are often applied to generalize the performance of deep learning models to previously unseen data (Connor et al., 2021). They can be either constructed from a set of data augmentation tools (e.g., rotation, flipping, and scaling) or learned through a set of Lie group operators that define directions of motion on the manifold. Both classes can be unified into motion-induced identity-preserving transformations by generalizing the untangling factor from a viewpoint only to motion-related variations. Broadly speaking, based on the observation that the identity of an object is temporally stable, identity-preserving transformations should include both microscale (e.g., saccadic-driven image translations) and macroscale (e.g., egomotion-driven clutter variability). Additionally, deformable objects such as faces and bodies pose additional challenges to invariant recognition, which calls for a recursive application of identity-preserving transformations (e.g., reentrant signaling, Edelman, 1993).

A closely related idea to manifold untangling is the learning of disentangled representations. For example, the GAN for disentangled representation learning (DR-GAN) (Tran et al., 2017) can take one or multiple images as input and explicitly output the pose code along with an arbitrary number of synthetic images. Such a GAN-based deep-generative model cleverly combines the pose code in the generator and the pose estimation in the discriminator into a closed loop. It can be interpreted as achieving tolerance by simultaneously resolving the uncertainty of identity and pose. It is mathematically equivalent to the maximum a posterior (MAP) estimation in the joint space of object identity and identity-preserving transformations (refer to Figure 4D in DiCarlo et al., 2012).

### 4.2. Invariant speech and language recognition

Unlike image data, speech signals are characterized by dynamic patterns in the temporal domain. Since language is unique to humans, language models serve as a strong supervisor in speech recognition. From words and phrases to paragraphs and part-of-speech, the principle of hierarchical organization has been widely studied in natural language processing. Computational maps in the auditory cortex share an organizational principle similar to that in the visual cortex (Krumhansl, 2001). Therefore, it is enlightening to understand invariant speech and language recognition from a manifold untangling perspective.

Compared to images, speech and language data are arguably less tangled due to the varying physical origin. From a manifold untangling perspective, embedding plays a more important role than flattening for speech and language data than for images. This difference is supported by the popularity of word embedding models [e.g., word2vec (Goldberg and Levy, 2014) and GloVE (Pennington et al., 2014)]. Even without any flattening, it is relatively easy to untangle the word manifold by embedding alone, as shown in recent work using two models of automatic speech recognition (ASR) models (Stephenson et al., 2019): convolutional neural network (CNN)-based (Kell et al., 2018) and Deep Speech 2 (DS2) (Amodei et al., 2016). The untangling of the word manifold has been clearly demonstrated by the increase in manifold capacity of both the ASR and DS2 models in later layers. A similar observation has been made for the popular language model (BERT) which is transformer-based (Mamou et al., 2020).

### 4.3. Perceptual straightening of video data

By contrast, video data has been much less studied than image or speech. Depending on the definition of object category, we can revisit several classical video processing tasks from a manifold untangling perspective. First, the class of natural video defines a manifold that is related to visual quality. The amount of perturbation (e.g., jittering artifacts) from the manifold of natural video is often correlated with the degradation of visual quality. One of recent works (Hénaff et al., 2019) has proposed a predictive coding hypothesis (Rao and Ballard, 1999)—that is, the temporal trajectories of visual input are perceptually straightened to make them more predictable. This hypothesis is consistent with the theory of manifold untangling because temporal straightening can be interpreted as a strategy of flattening the object manifold associated with the subspace of viewpoint. A key experimental finding from Hénaff et al. (2019) is that natural motion in video sequences corresponds to a flat trajectory in the perceptual space. Such a manifold flattening viewpoint seems to offer a quantitative framework for evaluating the performance of video stabilization techniques (Roberto e Souza et al., 2022).

Second, the concept of probabilistic appearance manifold has been introduced for video-based face recognition (FR) (Lee et al., 2003). In Lee et al. (2003), the local geometry of the non-linear appearance manifold (associated with varying poses) is approximated by standard PCA-based hyperplanes. Such a linear approximation of the pose manifold is conceptually simple, but its optimality is often questionable. The theory of manifold untangling offers a refreshing new perspective toward video-based FR—that is, one can flatten the pose manifold in the latent space (e.g., *W*+ in StyleGAN, Shen et al., 2020). After straightening the video of a given identity, one can interpret the warped video as augmented image observation by pose normalization. It follows that even simple fusion strategy, such as sum-rule, can be applied to the untangled video data. Note that such an idea of untangling manifolds can be easily generalized from the pose manifold to other facial attributes (e.g., age and expression).

Third, a dual problem with image-based object recognition is dynamic scene classification (Theriault et al., 2013) where the object category is semantically defined by the scene of video data. Learning the slowest feature with slow feature analysis (SFA) (Wiskott and Sejnowski, 2002), one can untangle the classes for different semantic categories. The key idea behind SFA is to learn invariant representations from transformation sequences, which is closely related to Laplacian eigenmaps (Sprekeler, 2011). From the perspective of manifold untangling, SFA can be interpreted as an alternative to selectivity and tolerance to learning invariance (Franzius et al., 2008). A similar idea has also found a successful application in the untangling of the manifold of motion for the recognition of human action (Zhang and Tao, 2012). One possible extension of SFA inspired by manifold embedding is to concatenate the learned SFA features from multiple modalities (e.g., color, SIFT, HOG); when motion information is represented by gait or skeleton, manifold flattening can be easily implemented by deformable shapes, Palafox et al., 2021).

## 5. Biological connections with sensory processing, motor control, and binding problem

### 5.1. Cortically local subspace untangling in ventral stream

How is manifold untangling achieved by the ventral stream of the visual cortex? In DiCarlo et al. (2012), it was hypothesized that the task is implemented recursively using a meta-job description at different layers. At each layer, the objective of a local group of neuronal population is to ensure that the output representation becomes less tangled than the input one, which gives the term “cortically local subspace untangling”. Two general classes of mechanisms are conceived to be relevant to the task of flattening manifolds: non-linear network architecture (Riesenhuber and Poggio, 1999; Serre et al., 2007) and identity-preserving transformations (Pagan et al., 2013; Mocz et al., 2021), which we will briefly review here.

In the hierarchical HMAX model for object recognition (Riesenhuber and Poggio, 1999), two classes of cells (simple vs. complex) are responsible for selectivity and tolerance operations, respectively. There exists a canonical circuit to model simple and complex cells in V1 (Kouh and Poggio, 2008) based on non-linear divisive normalization. Generally speaking, simple cells are modeled by AND-like or summation operators, which constructs some selective tuning for combinations of visual features; complex cells are modeled by OR-like or max-pooling operators, which achieve invariance/tore lance to variations in the visual stimuli (e.g., pose, location, and scale). HMAX model and convolutional neural networks (CNN) consist of several layers of alternating simple and complex cells, which can be interpreted as gradually untangling object manifolds (Brahma et al., 2015). However, unlike the convergent architecture in HMAX or CNN, the visual cortex is known for its divergent topology (Barlow, 2001) (consistent with the blessing of dimensionality).

The temporal continuity hypothesis states that “input patterns that occur close together in time tend to lead to similar output responses” (DiCarlo et al., 2012). Since an object's identity is temporally stable/continuous, retinal images of the same object naturally serve as training data for learning identity-preserving transformations. For example, it is well-known that inferotemporal cortex (IT) neurons are capable of responding similarly to the same object regardless of its retinal positions. This tolerance of spatial location can be explained away from the perspective of getting bootstrapped by the large number of saccadic-driven translation experiences of retinal images. Similar observations can be made with respect to the tolerance of the object's rotation but up to a certain angle. Meanwhile, the perirhinal cortex (PRH) is responsible for item memory, especially when representing familiar items; such familiarity with items can be interpreted as finer-grained untangling than position and rotation. In fact, the experimental results have confirmed that along with the flow of information from IT to PRH, the representation of the visual object becomes more untangled (Pagan et al., 2013).

### 5.2. Trajectory untangling in motor control

J. Gibson says that “we move because we see; we see because we move.” The dual view toward perception and motion inspires us to consider the problem of manifold untangling for the motor cortex as the dual for the visual cortex. In Russo et al. (2018) it has been observed that, unlike muscle activity, neural activity is structured in such a way as to avoid tangling, that is, similar neural activity patterns lead to dissimilar action patterns in the future (an object action-related counterpart of object recognition). How does the motor cortex encode muscle-like commands? Hypothesis about encoding of movement velocity or direction exists in the literature (e.g., Gallego et al., 2017); however, sophisticated tasks such as reaching and cycling (or more extended movements) suggest that neural activities are dominated by signals that are not muscle-like (therefore cannot be explained by velocity/direction coding) at the population level (Russo et al., 2018).

Based on the premise that the present network state strongly influences the future state, we conjecture that the objective of *trajectory untangling* is also recursively (although via hierarchical timescale instead of spatial scales) achieved by the motor cortex. Conceptually similar to the tangling in object recognition, the principle of trajectory untangling implies that two similar patterns of neural activity, observed as different moments, should not produce highly dissimilar action patterns in the near future. Violation of such principle often leads to trajectory tangling, a potential instability in the network dynamics of motor control. A key finding from the cycling experiment from Russo et al. (2018) is that “muscle-like signals are present, but are relatively modest ‘ripples that ride on top of larger signals that confer minimal tangling.”

The perspective of trajectory untangling is consistent with the closed-loop theory of motor learning (Adams, 1971). For closed-loop optimization, error feedback that plays a role in the reinforcement learning of simple movements can be interpreted as manifold projection. Trajectory untangling facilitates the task of closed-loop optimization by decomposing the movement into the knowledge of the result (trends) and the withdrawal of reinforcement (ripples). The learning procedure of motor skills is then abstracted as gradual untangling of trajectories in the latent space of motor control (Langdon et al., 2023). More recently, the problem of motor control has been studied more rigorously using the theory of dynamical systems. Motor learning on the neuronal population dynamics scale was shown to involve multiple learning mechanisms operating on different timescales (Vyas et al., 2020). Studies on motor learning have shown the benefit of forming motion memory from action observation (Mattar and Gribble, 2005; Stefan et al., 2005). More recently, it has been reported that (1) smooth mappings of experimental parameters onto flat neural manifolds can increase demixability (Kobak et al., 2016); and (2) neural networks with low-rank connectivities can produce demixed manifolds (Keemink and Machens, 2019).

### 5.3. From perceptual untangling to internal representation

**Figure 4:**
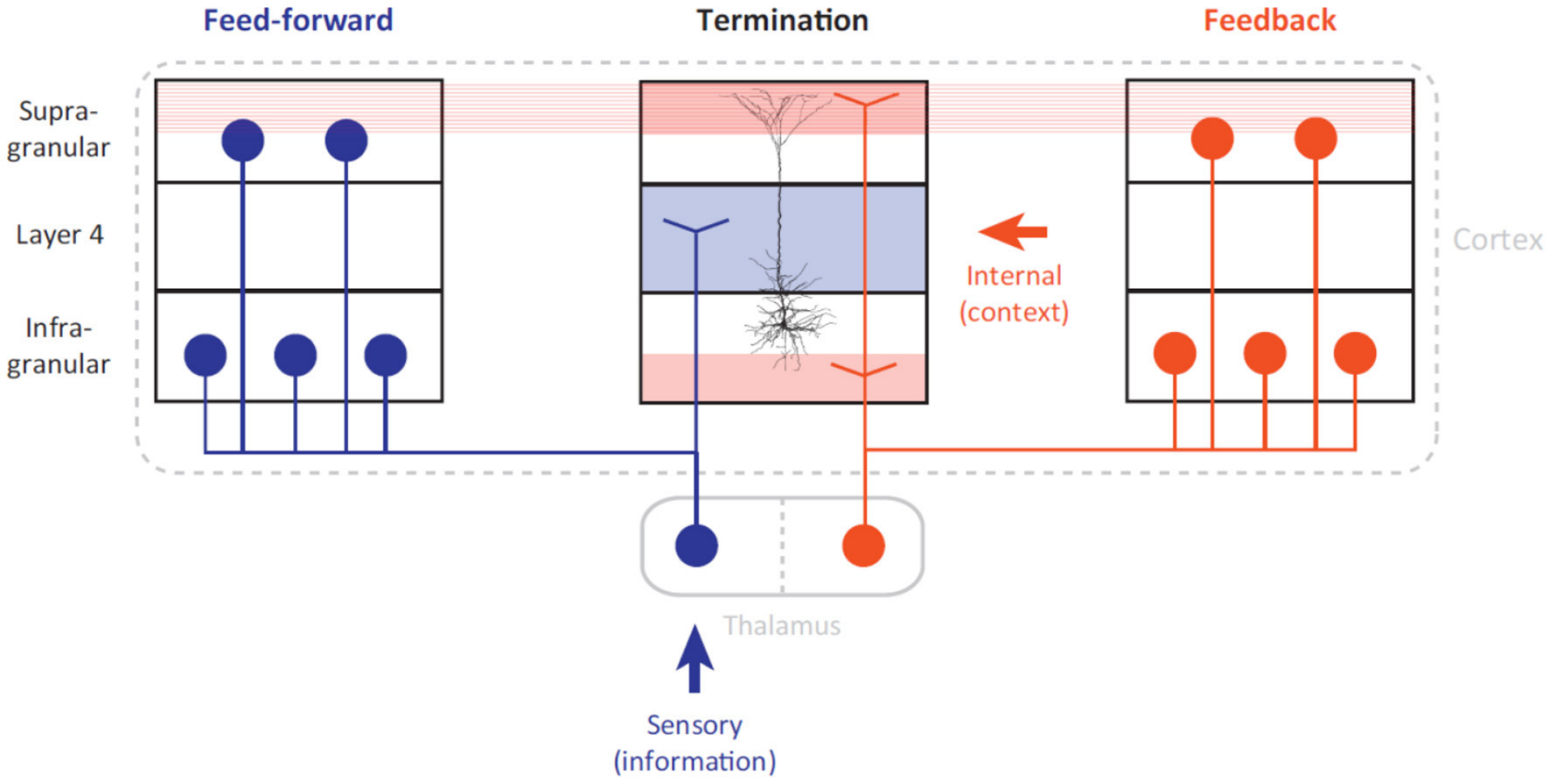
Long-range architecture of the cortex (cited from Larkum, 2013). The feed-forward stream (marked by the color blue) is driven by external information that influences the sensory apparatus. The feedback stream (marked by the color red) is driven by an internal representation built from previous experiences. We conjecture that the feedforward and feedback streams can be geometrically interpreted as manifold embedding and projection, respectively.

According to Helmholtz (Lee, 2015), the fundamental role of the neocortex is to construct an internal representation of the external environment. Mirroring of the physical world in the primate brain is achieved by the constant interaction between the sensory and motor cortex. It has been suggested that the organizational principle of the cortex, regardless of object recognition or motor control, shares a similar association mechanism at the cellular level (Larkum, 2013). As shown in Figure 4, pyramidal neurons play the role of coupling feed-forward with feedback streams that are driven by external stimuli and internal representation, respectively. This association mechanism at the cellular level succinctly explains the advantage of the cortical hierarchy, with its structured terminations at different layers. It also offers a plausible explanation for how neuronal populations in various areas can be “bound” instantaneously to represent tangled features.

Thalamo-cortical interaction must occur simultaneously in both feed-forward and feedback streams to support the predictive coding hypothesis in the visual cortex (Rao and Ballard, 1999). A feedforward visual stream transmits external stimuli information to higher cortical areas through manifold untangling; pyramidal neurons act as associative elements that detect coincidences between present stimuli and experience (internal representation). Then, the feedback stream serves as the prediction coding scheme (Rao and Ballard, 1999) of the cortex that determines the firing of pyramidal neurons. Given that 90% of the synaptic input to layer-1 (L1) are from long-range feedback connections, the backpropagation-activated coupling (BAC) (Larkum, 2013) firing mechanism of pyramidal neurons has been shown to bridge the feedforward (manifold untangling) and feedback (manifold projection) streams.

The bridging of feed-forward and feedback streams is consistent with the new perspective of how the binding problem was solved by base grouping (feed-forward processing) and incremental grouping (feedback connection) (Roelfsema, 2023). It was argued that the distribution of visual attention is largely determined by motor control or action planning. More specifically, the process of selecting objects for perceptual processing and object recognition is coupled with that of providing the information necessary for motor action through a single attentional mechanism (Deubel and Schneider, 1996). From the manifold untangling perspective, feedforward processing is responsible for the tuning of neurons to features and base groupings; while feedback connections enhance the firing rates of to-be-grouped features through manifold projection. Manifold untangling facilitates the solution to the binding problem by re-representing different sensory stimuli into groupable features (e.g., position, size, and pose). Along this line of reasoning, enhancing firing rates alone (no need for neural oscillation and synchrony, Von Der Malsburg, 1994) is sufficient for the binding or integration of groupable features from different modalities.

Finally, hippocampus, seated on the top of neocortical pyramid, is responsible for storing memories of specific events and places. It plays a key role in constructing an internal representation of the external world, which involves integrating information from different sensory modalities and binding them into a coherent memory. The dentate gyrus (DG), a subregion of the hippocampus, interacts with the other subregions of the hippocampus (e.g., including the CA1 and CA3 regions) to form a functional network that is critical for memory processing and retrieval. In feed-forward processing, the entorhinal cortex sends sensory information from the neocortex to the dentate gyrus, which then processes and integrates the information with other sensory inputs in the hippocampus. Manifold unfolding is implemented by DG which performs the decorrelation and sparsification of input signals by projecting to higher-dimensional space. In feedback processing, manifold projection simply projects the stored information back to the neocortical regions, which is consistent with hippcampal index theory (Teyler and DiScenna, 1986).

## 6. Conclusions

It has been hypothesized that through neuronal population dynamics, the neocortex solves the problem of object recognition via perceptual untangling. We formulate the problem of manifold untangling as an abstraction of object recognition in this paper. Two complementary approaches to untangle an object manifold are presented: embedding (selectivity-promoting) and flattening (tolerance-promoting). We have discussed two classes of embedding strategies (generalized kernel method and hierarchical sparse coding) as well as flattening strategies (identity-preserving transformation and decision boundary smoothing). Under the framework of manifold unfolding, we present a unified interpretation of multiview image recognition, invariant audio/language recognition, and perceptual straightening of video. Finally, the theory of manifold unfolding is connected with the literature of neuroscience, which demonstrates the biologically plausible implementation of perceptual untangling.

Future works require the development of experimentally or computationally testable hypotheses or models built upon the theory of manifold untangling. Deep neural networks have shown to demonstrate some interesting manifold disentangling properties in Brahma et al. (2015) and Horan et al. (2021). However, existing neural architectures such as convolutional neural networks do not exactly match the divergent topology of neocortex—namely, there are a lot more neurons and synapses in the higher levels than those in the lower levels. The class of over-parameterized neural networks (Du et al., 2019) and over-complete representations (Chen et al., 2013) arguably better reflects the organizational principles of ventral stream processing. Therefore, we believe that the theory of manifold untangling can be more easily falsified from the class of over-parameterized models. For example, recently developed large vision models (e.g., scaling vision transformers, Zhai et al., 2022) might serve as a promising proxy for studying object recognition by ventral stream processing.

## Data availability statement

The original contributions presented in the study are included in the article/supplementary material, further inquiries can be directed to the corresponding author.

## Author contributions

Both authors listed have made a substantial, direct, and intellectual contribution to the work and approved it for publication.
